# Serum cystatin C is increased in acute spinal cord injury: a multicentre retrospective study

**DOI:** 10.1038/s41393-019-0360-7

**Published:** 2019-10-04

**Authors:** JinYuan Zhang, RuoTing Ding, QingZhang Xian, ZhiKun Wang, ZhongYuan Liu, JinCheng Yang, JianTing Chen

**Affiliations:** 1 Department of Spinal Surgery, Nanfang Hospital, Southern Medical University, Guangzhou, China; 2 Department of Orthopedics, Panyu Hospital of Traditional Chinese Medicine, Guangzhou, China; 3 Department of Spinal Surgery, Dongguan Third People’s Hospital, Dongguan, China; 4 Department of Spinal Surgery, General Hospital of Guangzhou Military Command, Guangzhou, China

**Keywords:** Predictive markers, Trauma

## Abstract

**Study design:**

A multicentre retrospective study.

**Objective:**

A multicentre retrospective study was performed to observe the changes in serum cystatin C (CysC) levels in patients with acute spinal cord injury (SCI).

**Setting:**

Four hospitals in China.

**Methods:**

Over a 5-year study period, the CysC, creatinine (Cr), and blood urea nitrogen (BUN) levels of people who had incurred SCI in the preceding 7 days were collected and compared with those of people with limb fracture (LF) who were matched for injury time and gender. People with SCI also were grouped by injury duration, ASIA Impairment Scale (AIS) grade and the presence or absence of steroid therapy and compared each day.

**Results:**

Three hundred and twenty-three samples from people with SCI were retrospectively collected; their mean serum CysC levels were significantly higher than those of people with LF (*p* < 0.001); No significant difference was observed in Cr or BUN levels between the two groups (*p* > 0.14). CysC levels increased on the second day, peaked on day 3, and returned to normal on day 5. The more severely injured individuals had higher CysC levels. Steroid therapy or not had no influence for CysC levels.

**Conclusion:**

CysC levels are increased in patients with acute SCI, possibly as a direct result of injury. Serum CysC is a potential biomarker of SCI.

## Introduction

Spinal cord injury (SCI) leads to immediate cell death and tissue damage, followed by multiple biochemical changes, including inflammatory response, oxidative stress, and cell apoptosis [1]. However, there are currently no biomarkers in common clinical use to predict the prognosis or observe neurological changes after SCI, especially for patients under sedation whose muscle strength and sensation are difficult to assess [2].

Cystatin C (CysC) is a marker of kidney dysfunction [3, 4]. A high level of this biomarker has been observed in the cerebrospinal fluid (CSF) of people who have had a traumatic brain injury, including both children and adults. In addition, many animal experiments have shown that CysC protects the brain from secondary injury by various mechanisms [5, 6]. Furthermore, a study of acute cerebral stroke found that CysC was an independent predictor of infarct size and haemorrhage volume [7]. CysC was strongly associated with acute ischaemic stroke and was an independent predictive marker of acute ischaemic stroke [8].

Although most studies of CysC have focused on the brain, some studies have reported CysC levels in people with chronic SCI [9–11]. CysC has been shown to be more valuable than creatinine (Cr) as an indicator of renal function in SCI because of the stability of the CysC level, which is independent of gender, age, and muscle mass [12]. No studies have investigated CysC in acute SCI patients and it is still unknown how serum CysC levels change in acute SCI. Therefore, the aim of this study was to observe serum CysC levels in people who are in the acute stage of SCI. We hoped to identify a relationship between CysC levels and SCI through this retrospective multicentre study, which may provide an economical biomarker for widespread clinical use.

## Methods

**Fig. 1:**
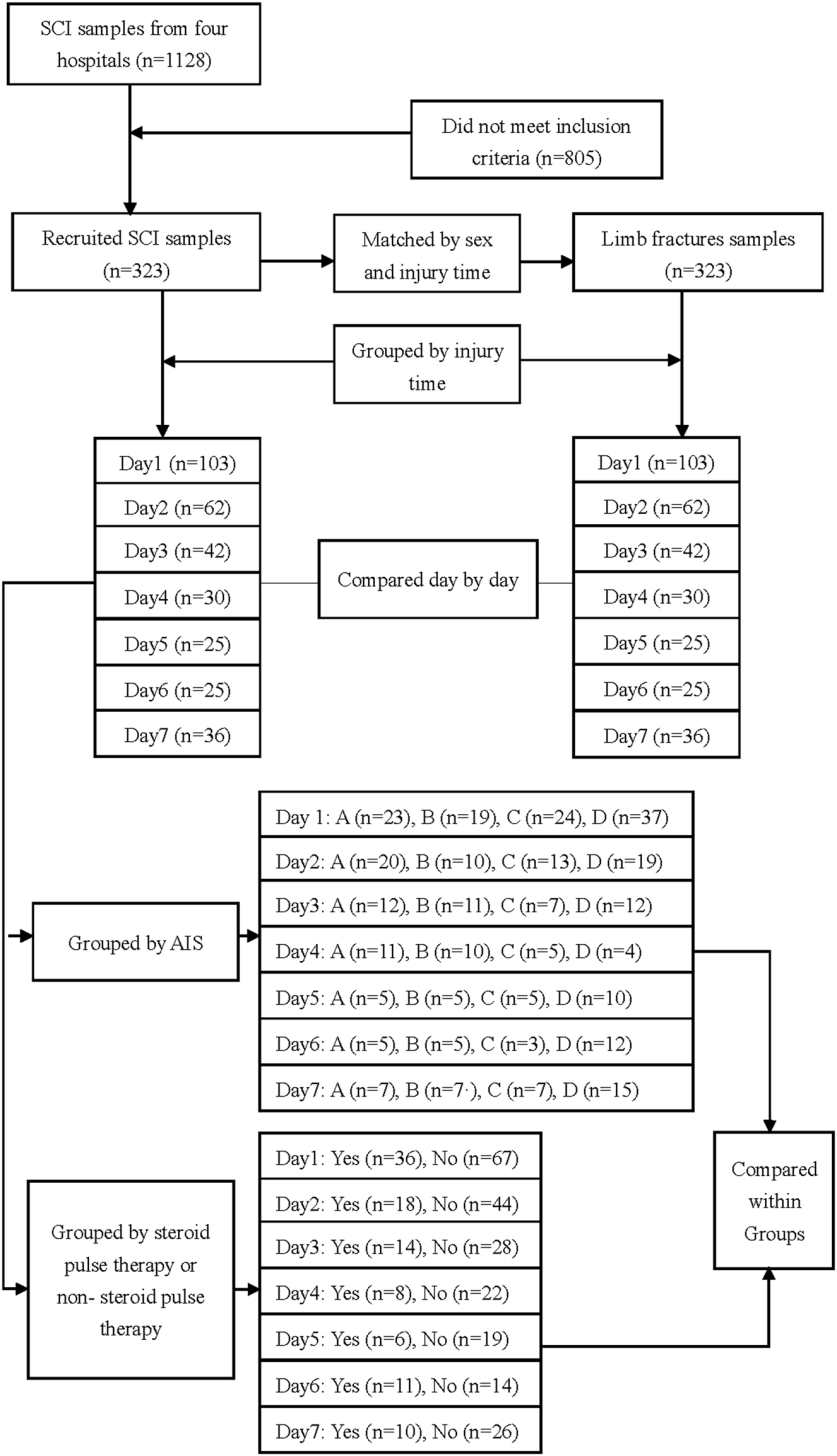
The flow diagram of the study. Three hundred twenty-three samples were reviewed from 273 people with SCI matched with LF samples for daily comparisons. In addition, we also grouped and compared samples by AIS grades and the presence or absence of steroid therapy

The CysC levels of people with SCI and people with limb fracture (LF) were retrospectively collected from medical records to assess the impact of CysC levels on acute SCI (7 days after injury). The data were gathered from four hospitals (Nanfang Hospital, Panyu Hospital of Traditional Chinese Medicine, Dongguan Third People’s Hospital, General Hospital of Guangzhou Military Command) for patients admitted between 1st August 2011 to 6th January 2017. People with LF were matched 1:1 to people with SCI in the same hospitals by injury time and gender. The LF group was free of SCI. Both groups were treated with general therapy, including wound care, haemostasis, rehydration, analgesia, nutritional support, and bed rest or limb immobilization. The same exclusion criteria were applied to both the acute SCI group and the LF group, namely: other types of stroke or serious organic diseases of the spine, neurodegenerative diseases, metabolic diseases, cancer, serious heart diseases, renal diseases, liver diseases, haematological disorders, and infective or inflammatory disorders. The flow diagram is shown in Fig. 1.

The CysC, Cr, and BUN levels were measured at the four hospitals using BECKMAN COULTER AU5800 (BECKMAN COULTER, CA, USA), HITACHI 7600 (HITACHI, Tokyo, Japan), Olympus AU2700 (Olympus Corporation, Tokyo, Japan), and Roche COBAS-C-702 (Roche, Basel, Switzerland) automatic analysers, with the reference intervals as follows: CysC, 0.63–1.25 mg/L; Cr, males 53–106 μmol/L and females 44–97 μmol/L; and BUN, 2.8–7.2 mmol/L. We collected the data from recorded test reports. All the test instruments were standardized and supervised by the government. The time from injury to blood sampling was recorded for each case.

The American Spinal Injury Association (ASIA) Impairment Scale (AIS) grades were collected from the records of each case. AIS scores were measured within 24 h of hospitalization. The use of steroid therapy or non-steroid therapy was also recorded.

Data are presented as the mean ± SD, and comparisons were made using paired *t*-tests and independent samples *t*-tests. Statistical analyses were performed with SPSS 13.0 for Windows (SPSS, Chicago, USA).

## Results

Three hundred and twenty-three samples which came from 273 people with SCI were collected from a total of 1128 samples; 323 corresponding samples with LF were matched with them. Among the participants with SCI, there were 258 male samples and 65 female samples; the proportions were the same in the LF group. The mean (SD) age of the SCI group was 44.8 (12.1) years, and the LF group was 44.7 (12.3). The most common cause of SCI and LF was falls. The SCIs were mostly at the cervical level and the LFs were mostly of the femur (Table 1). Individual data for all outcomes can be seen in Supplementary Material for primary data.

**Table 1:** The characteristics of SCI samples

Variable	SCI		Limbs fractures		*p* value
Mean age	44.8 ± 12.1		44.7 ± 12.3		0.072
Samples	323		323		
Male	258		258		
Female	65		65		
Injury area					
	Cervical	269	Femoral	125	
	Cervical–thoracic	15	Tibiofibula	97	
	Thoracic	23	Calcaneal	26	
	Thoracic–lumbar	2	Humerus	20	
	Lumbar	14	Others^a^	55	
Aetiology					
	Fall	193	Fall	162	
	Traffic accidents	99	Traffic accidents	120	
	Violence	17	Violence	19	
	Struck by object	12	Struck by object	19	
	Spinal massage	2	Others^b^	3	

The characteristics of SCI samples. These samples were matched 1:1 to samples with LF by injury time and sex. In addition, there was no statistically significant difference in age

^a^The injured areas included the patella, ulna, radius, and clavicle, as well as multiple injuries of the hand or foot

^b^The aetiologies were sports, cuts, and gunshots

The mean CysC concentration was significantly higher in the SCI group than in the LF group. However, there was no significant difference in Cr or BUN concentrations between the two groups (Table 2).

**Table 2:** Comparison of concentrations of CysC, Cr, and BUN levels between the SCI and LF groups (mean ± SD)

	SCI (*n* = 324)	Limbs fracture (*n* = 324)	*r* value	*p* value
CysC	0.857 ± 0.220	0.774 ± 0.151	0.062	<0.001
Cr	71.52 ± 17.749	69.481 ± 25.833	0.117	0.245
BUN	5.649 ± 1.817	5.443 ± 1.643	0.010	0.143

Paired *t*-tests were used for the comparisons. The mean CysC level of the SCI group was significantly higher than that of the LF group, but there was no statistically significant difference in Cr or BUN levels between the groups.

**Fig. 2:**
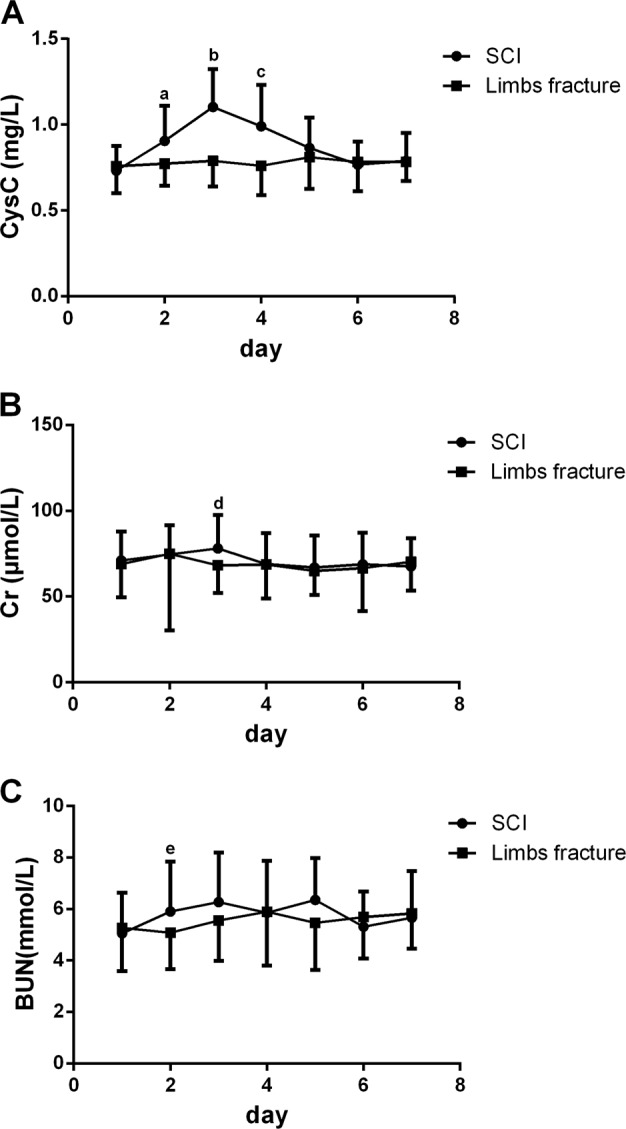
The mean CysC, Cr, and BUN levels of the SCI and LF groups on each of the first 7 days. CysC levels increased on the second day, reached their peaked on day 3, and returned to normal on day 5. **a** The mean CysC levels of the SCI subgroups were significantly higher than those of the LF subgroups on days 2 (^a^*p* < 0.001), 3 (^b^*p* < 0.001), and 4 (^c^*p* < 0.001). **b** The Cr levels of the SCI group were significantly higher than those of the LF group on day 3 (^d^*p* = 0.06). **c** The BUN levels of the SCI group were significant higher than those of LF group on day 2 (^e^*p* = 0.06)

The SCI and the LF groups were divided into seven subgroups according to time since injury (Fig. 1). CysC, Cr, and BUN levels were compared with those of the matched LF group. CysC levels increased on the second day, reached their peak on day 3, and returned to normal on day 5. The mean CysC levels of the SCI subgroups were significantly higher than those of the LF subgroups on days 2 (*p* < 0.001), 3 (*p* < 0.001), and 4 (*p* < 0.001). The Cr levels of the SCI group were significant higher than those of the LF group on day 3 (*p* = 0.006), and the BUN levels of the SCI group were also significant higher than those of the LF group on day 2 (*p* = 0.006), but there were no significant differences between the Cr and BUN levels on the other days (Fig. 2 and Table 3).

**Table 3:** Comparison of CysC, Cr, and BUN levels between the SCI and LF subgroups on each seven days (mean ± SD)

	CysC			Cr			BUN		
	SCI	LF	*p* value	SCI	LF	*p* value	SCI	LF	*p* value
Day 1	0.732 ± 0.143	0.758 ± 0.158	0.181	71.016 ± 16.924	68.897 ± 19.158	0.392	5.054 ± 1.585	5.274 ± 1.681	0.341
Day 2	0.905 ± 0.207	0.772 ± 0.129	<0.001	74.532 ± 17.033	74.948 ± 44.665	0.943	5.903 ± 1.94	5.083 ± 1.415	0.006
Day 3	1.104 ± 0.221	0.79 ± 0.15	<0.001	78.014 ± 19.418	68.131 ± 16.087	0.006	6.271 ± 1.926	5.556 ± 1.567	0.074
Day 4	0.991 ± 0.241	0.761 ± 0.171	<0.001	68.927 ± 17.949	68.7 ± 19.85	0.961	5.854 ± 2.014	5.892 ± 2.092	0.947
Day 5	0.864 ± 0.177	0.81 ± 0.185	0.274	66.884 ± 18.846	64.96 ± 13.959	0.654	6.354 ± 1.636	5.458 ± 1.826	0.087
Day 6	0.768 ± 0.133	0.784 ± 0.172	0.660	68.8 ± 18.528	66.536 ± 25.124	0.704	5.312 ± 1.365	5.685 ± 1.607	0.436
Day 7	0.789 ± 0.162	0.783 ± 0.111	0.847	67.539 ± 16.404	70.353 ± 16.843	0.442	5.666 ± 1.815	5.832 ± 1.372	0.587

Paired *t*-tests were used for the comparisons. The results were also shown in Fig. 2

**Fig. 3:**
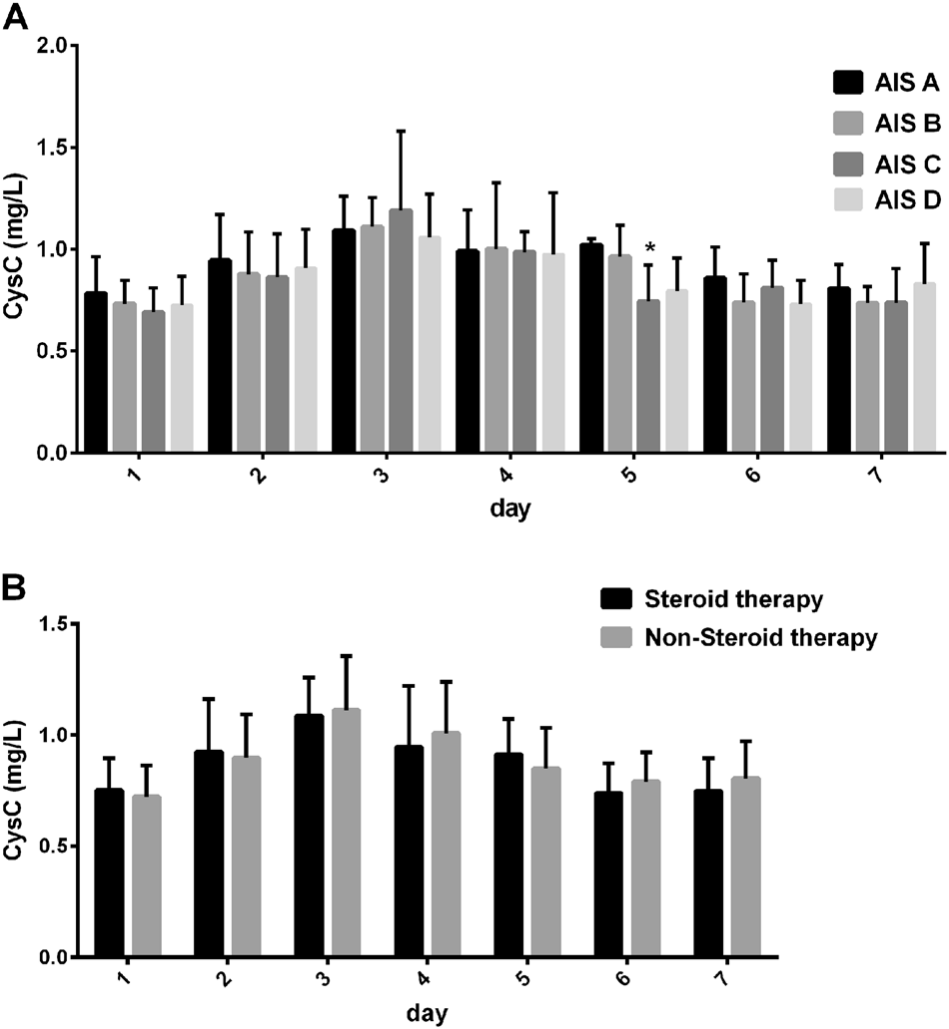
Comparison of CysC levels by AIS grade and presence or absence of steroid therapy on each of the first 7 days. **a** SCI samples were regrouped by AIS grade, and the CysC levels were compared within each day. No significant differences were found except on the fifth day. The CysC levels of the AIS A group were significantly higher than those of the AIS C group (*p = 0.029); **b** SCI samples were regrouped by whether they had received steroid therapy, and these subgroups were compared within each day. No significant differences were found among the subgroups (*p* > 0.3)

People with SCI people were grouped by AIS grade (Fig. 1). Multiple *t*-tests with a Bonferroni correction were applied, and CysC levels of the AIS A group was higher than those of the AIS C and D groups (*p* < 0.047). Given the possible effect of time, the four groups by AIS grade were compared within each day separately. There were still no significant differences in CysC levels on the first, second, third, fourth, sixth, or seventh day. On day 5, the mean CysC concentrations of the AIS A group were higher than those of the AIS C group (*p* = 0.043); (Fig. 3).

Furthermore, the SCI group who had undergone steroid therapy was compared with those who had not received steroid therapy (Fig. 1). The mean CysC levels of the steroid therapy group were lower than that of the non-steroid-pulse group, but the difference was not significant (*p* = 0.701). Recipients and non-recipients were also compared each day. We found no significant differences in CysC levels among these subgroups (*p* > 0.3) (Fig. 3).

## Discussion

To the best of our knowledge, the present study is the first to focus on CysC levels in acute SCI. The mean CysC levels of people with SCI were closer to those of people with brain injury but lower than levels of those with renal insufficiency. In people with ischaemic stroke, the mean (SD) levels of CysC in the acute stage have been reported as 1.067 (0.298) mg/L [7], which is lower than the levels we found on the third day. These previous articles discussed only acute-stage CysC levels, not peak levels. However, in people with renal dysfunction, the mean CysC levels were 2.5(0.6) mg/L [13], which is higher than the levels in the samples with SCI. Other studies recruited healthy people as controls, and their average CysC concentrations were 0.793 (0.110) mg/L [7] and 0.77 (0.10) mg/L [14], which are similar to those of the LF group. CysC levels did not appear to be increased in LF samples. The third day after SCI is a critical point in injury progression, as the damaged area reaches a maximum [15], and the apoptotic index also peaks [16].

In addition to SCI, TBI, and kidney disease, other factors can promote upregulation of serum CysC. Individuals with hyperthyroidism also have high CysC levels [17], and cancer, hypertension, cardiovascular disease, and rheumatoid arthritis can interfere with the secretion of CysC. Individuals with these diseases were excluded from the present study. Glucocorticoid treatment is another important factor, which is why patients with and without steroid therapy were compared. High-dose methylprednisolone was commonly used previously, but this practise was controversial [18]. Therefore, not all participants received this treatment. In this study, no significant differences in CysC levels were found between people with and without steroid therapy; CysC levels did not appear to be associated with this therapy, even when corresponding time points were compared. Two studies showed that glucocorticoids could increase CysC levels. One experiment added dexamethasone to HeLa cells, which increased CysC levels [19]. However, HeLa cells are not central nervous system cells. The other study was a clinical trial, which examined steroid therapy for five idiopathic nephrotic syndromes of childhood and demonstrated that serum CysC concentrations remained unaffected [20]. In another study, CysC levels were found to be higher in the CSF of people with lumbar disk herniation than in that of healthy people recovering from fractured lower limbs, and the authors concluded that CysC levels were associated with pain [21]. Because we recruited people with LF who were also in pain, our results suggest that CysC levels have little relationship with non-neuropathic pain. The metabolic rates of SCI and LF people were altered by the hospital treatments, so a similar condition could be occurring with respect to pain. Ischaemia/hypoxia was established in the damaged area of the spinal cord, and the nutritional deprivation environment may cause CysC levels to increase.

To our knowledge, currently there are no biomarkers in common clinical use to predict the prognosis or observe neurological changes following SCI [2], especially in participants who are sedated, making testing of motor function and sensation difficult. A biomarker may be helpful to determine the severity of the SCI and its prognosis, especially in hospitals that may lack expensive equipment, such as MRI scanners [22]. CysC levels have been widely used in clinics, and CysC may be an economical biomarker for SCI based on the premise of excluding certain primary diseases.

We collected Cr levels, BUN levels, and AIS grades. Although Cr levels increased on day 3 and BUN levels were increased on day 2, these levels were normal on most other days, suggesting that glomerular function may not be damaged after SCI; [23] thus, CysC may not be an appropriate indicator of renal function in acute SCI, or its levels at least may need to be adjusted. When samples were grouped by AIS grades, CysC levels of the AIS A group were higher than those of the AIS C and D groups. In an analysis spanning the first 7 days, the AIS A group had higher CysC levels than the AIS C group on day 5. The CysC levels seemed to be related to SCI severity. However, the AIS A and C groups only included five persons each, thus limiting our ability to generalize these findings. AIS grades are related to the completeness of the SCI and thus only reflect function, not the damaged area. We were still unable to confirm that CysC levels ware associated with AIS grades.

As the incidence of SCI is rather low, retrospective analysis of data collected as part of routine care is a feasible and economical way to study SCI in humans. However, this design has some limitations. For instance, the body mass index was not analysed, due to missing data. In addition, we did not investigate gender, age or body condition, as CysC levels had already been established as independent of those factors [24]. Because CysC levels most likely are influenced by interindividual differences, simply collecting different participants’ serum CysC levels for statistical analysis is not a strong research design. In addition, a retrospective study cannot show causality, so a cohort study is now needed. Most experiments on CysC have chosen healthy people as their control group. However, people with LF were in similar situations, and the two groups were matched with respect to time since injury; our study may also be the first to describe the daily trends in CysC levels. The very acute stage of SCI is 15 days, but we collected data only from the first 7 days. Initially, data were collected for all 15 days, but after the seventh day, CysC levels were no longer elevated; therefore, it was more economical to limit the observation period to 7 days.

In summary, serum CysC levels increase peaks on day 3 and then returns to normal levels on day 5. These changes in CysC levels are associated with SCI, and CysC may be a potentially useful biomarker of SCI severity.

## Supplementary information

The legend for the supplementary material

The primary data

## Data Availability

All data generated or analysed during this study are included in this published article and its supplementary information files.
