# Supplementary material for: Serum cystatin C is increased in acute spinal cord injury: a multicentre retrospective study
Source: Spinal Cord. 2019 Oct 4;58(3):341–7. doi: 10.1038/s41393-019-0360-7 (PMC7062626; doi:10.1038/s41393-019-0360-7)
Supplement: Supplementary file 1 — The legend for the supplementary material [file 41393_2019_360_MOESM1_ESM.docx]

All the primary data of this research including sex, age, draw blood date, injury time, injury segments, CysC，Cr, BUN, C-reactive, have MRI or not, AIS, steroid therapy or not, cause of disease.
